# Identification of potential molecular targets for the treatment of cluster 1 human pheochromocytoma and paraganglioma via comprehensive proteomic characterization

**DOI:** 10.1186/s12014-023-09428-7

**Published:** 2023-09-25

**Authors:** Ondrej Vit, Pavel Talacko, Zdenek Musil, Igor Hartmann, Karel Pacak, Jiri Petrak

**Affiliations:** 1 BIOCEV, First Faculty of Medicine, Charles University, Vestec, 25250 Czech Republic; 2 Proteomics Core Facility, Faculty of Science, BIOCEV, Charles University, Vestec, 25250 Czech Republic; 3 Institute of Biology and Medical Genetics, First Faculty of Medicine, Charles University and General University Hospital, Prague, 12800 Czech Republic; 4 Department of Urology, University Hospital Olomouc and Faculty of Medicine and Dentistry, Palacky University Olomouc, Olomouc, 77900 Czech Republic; 5 Section on Medical Neuroendocrinology, Eunice Kennedy Shriver National Institute of Child Health and Human Development, NIH, Bethesda, MD 20892 USA

**Keywords:** Pheochromocytoma, Paraganglioma, Neuroendocrine tumors, Integral membrane proteins, Membrane proteomics, Drug targets, Tumor imaging, Therapy

## Abstract

**Background:**

Pheochromocytomas and paragangliomas (PPGLs) are rare neuroendocrine tumors. New drug targets and proteins that would assist sensitive PPGL imagining could improve therapy and quality of life of patients with PPGL, namely those with recurrent or metastatic disease. Using a combined proteomic strategy, we looked for such clinically relevant targets among integral membrane proteins (IMPs) upregulated on the surface of tumor cells and non-membrane druggable enzymes in PPGL.

**Methods:**

We conducted a detailed proteomic analysis of 22 well-characterized human PPGL samples and normal chromaffin tissue from adrenal medulla. A standard quantitative proteomic analysis of tumor lysate, which provides information largely on non-membrane proteins, was accompanied by specific membrane proteome-aimed methods, namely glycopeptide enrichment using lectin-affinity, glycopeptide capture by hydrazide chemistry, and enrichment of membrane-embedded hydrophobic transmembrane segments.

**Results:**

The study identified 67 cell surface integral membrane proteins strongly upregulated in PPGL compared to control chromaffin tissue. We prioritized the proteins based on their already documented direct role in cancer cell growth or progression. Increased expression of the seven most promising drug targets (CD146, CD171, ANO1, CD39, ATP8A1, ACE and SLC7A1) were confirmed using specific antibodies. Our experimental strategy also provided expression data for soluble proteins. Among the druggable non-membrane enzymes upregulated in PPGL, we identified three potential drug targets (SHMT2, ARG2 and autotaxin) and verified their upregulated expression.

**Conclusions:**

Application of a combined proteomic strategy recently presented as “Pitchfork” enabled quantitative analysis of both, membrane and non-membrane proteome, and resulted in identification of 10 potential drug targets in human PPGL. Seven membrane proteins localized on the cell surface and three non-membrane druggable enzymes proteins were identified and verified as significantly upregulated in PPGL. All the proteins have been previously shown to be upregulated in several human cancers, and play direct role in cancer progression. Marked upregulation of these proteins along with their localization and established direct roles in tumor progression make these molecules promising candidates as drug targets or proteins for sensitive PPGL imaging.

**Supplementary Information:**

The online version contains supplementary material available at 10.1186/s12014-023-09428-7.

## Introduction

Pheochromocytomas and paragangliomas (PPGLs) are rare neuroendocrine tumors with an incidence rate of approximately 0.46 per 100,000 person-years for pheochromocytomas and 0.11 per 100,000 person-years for paragangliomas. While pheochromocytoma (also called adrenal paraganglioma) arises from chromaffin tissues of the adrenal medulla, paragangliomas arise from chromaffin cells of the sympathetic (thoracic, abdominal, and pelvic regions) or parasympathetic (head and neck region) paraganglia. Sympathetic PPGLs are typically secretory (e.g., catecholamine release) in nature and often associated with life-threatening complications. Up to 10–20% of PPGLs can develop metastases, mainly in bones, liver, lungs and lymphatic nodes [1]. PPGLs exhibit a high degree of heritability, with > 40% of affected patients carrying a germline pathogenic variant and 30% carrying a somatic pathogenic variant. To date, pathogenic variants or gene fusions in > 20 susceptibility genes associated with the development of PPGLs have been identified [1]. Based on the underlying mutations, expression profiles, and methylation status, PPGLs are divided into three molecular subtypes: pseudohypoxia-related (cluster 1), kinase signaling-related (cluster 2), and Wnt-related (cluster 3) PPGLs [2].

**Cluster 1** (pseudohypoxia-related) PPGLs represent two groups of tumors that are driven either by pathogenic variants in genes of the tricarboxylic acid (TCA) cycle (*SDHA*, *SDHB*, *SDHC*, *SDHD*, *SDHAF2*, *FH*, *IDH1/2*, *MDH2*) sometimes outlined as cluster 1 A, or by pathogenic variants in VHL/EPAS1-related genes (*PHD1/2*, *VHL*, *HIF2A/EPAS1*, *IRP1*) characterized as cluster 1B. Cluster 1 PPGLs represent high-risk tumors with a metastatic risk of 5–35% depending on the mutated gene; the risk is highest in *SDHB*-mutated PPGLs [3]. Cluster 1 PPGLs represent roughly 25–35% of all PPGLs.

**Cluster 2** PPGLs (roughly 50–60% of all PPGLs) comprise tumors harboring pathogenic variants in genes associated with tyrosine-kinase signaling (*RET*, *NF1*, *HRAS*, *TMEM127*, *MAX*, *FGFR1*, *MET*, *MERTK*, *BRAF* and *NGFR*). Cluster 2 PPGLs present frequently as bilateral pheochromocytomas which are not highly prone to metastatic progression (metastatic risk ranges between 2 and 11%) [4].

**Cluster 3** PPGLs are associated with the *MAML3* fusion gene and somatic pathogenic variants in *CSDE1*. These tumors and extremely rare, representing only 5–10% of all PPGLs [1].

In addition to the three well-defined clusters, there is a poorly characterized group of roughly 20–30% PPGLs, in which no germline or somatic pathogenic variants are observed in the known PPGL susceptibility genes. Hereafter, we refer to these tumors as **unassigned** PPGLs.

PPGLs can only be cured by complete removal via surgery. Metastases are diagnosed either at the initial presentation or many years after the first presentation. As there is no reliable histopathological or other type of marker to predict the tumor behavior, close follow-up is crucial for this disease [5]. The natural course of metastatic disease varies from very indolent (stable disease without any treatment for many years) to very aggressive (survival < 1 year). Despite some progress in treatment strategies, such as peptide receptor radionuclide therapy, there is no effective and specific treatment strategy available for patients with metastatic or inoperable PPGLs [1], represented by high-risk cluster 1 PPGLs. Therefore, there is an urgent need for new tumor imaging markers and specific drug targets for high-risk PPGLs.

Integral membrane proteins (IMPs) uniquely expressed or markedly upregulated on the cell surface of tumor cells, represent excellent drug targets. CD20, CD30, HER2, VEGFR1-3, somatostatin receptor, and PSMA can be mentioned as great examples. Cell surface protein-targeted radio-ligands, low molecular weight (LMW) inhibitors, therapeutic blocking antibodies, antibody conjugates, bispecific engagers, and CAR-T cells are the landmarks of the modern anti-cancer armamentarium. Similarly, intracellular proteins with enzymatic or regulatory activities critical for cancer cell proliferation, survival, or metastasis represent attractive drug targets, as specific LMW inhibitors can be used to disrupt their activity. Such proteins can be exemplified by the proteasome, PPAR1–2, estrogen receptor, or ribonucleotide reductase.

To identify new proteins that can be used as potential targets for PPGL therapy and/or tracers for tumor imaging, especially for high-risk cluster 1 PPGLs, we conducted detailed proteomic analysis of 22 well-characterized human PPGL and normal chromaffin tissue samples from the adrenal medulla. A standard quantitative proteomic analysis of tumor tissue was accompanied by specific membrane proteome-aimed methods, namely N-glycopeptide capture using lectin affinity [6] or hydrazide chemistry [7] and enrichment of membrane-embedded hydrophobic transmembrane segments [8]. The parallel application of these complementary methods, recently presented as a “Pitchfork strategy,” enables the deep quantitative profiling of human membrane proteomes, without sacrificing the information on soluble non-membrane proteins [9].

## Materials and methods

Unless specified otherwise, all reagents were purchased from Merck Life Science (Czech Republic).

### Patients and samples

In total, 22 PPGL samples were included in this study. These samples included 8 primary PGLs (paragangliomas), 11 primary PHEOs (pheochromocytoma), 1 recurrent PHEO, 1 recurrent and metastatic PGL, and 1 metastatic PGL. PPGLs included in this study were surgically resected from enrolled patients and evaluated under a protocol approved by the Eunice Kennedy Shriver National Institute of Child Health and Human Development, NIH, USA Institutional Review Board (ClinicalTrials.gov Identifier: NCT00004847). Informed consent was obtained from all patients for clinical, genetic, biochemical, and imaging studies performed as part of the investigation. This study was conducted in accordance with the principles of the Declaration of Helsinki. Tumors were resected between August, 2006 and December, 2017, and all were histopathologically confirmed as PHEOs or PGLs by the NCI Laboratory of Pathology (Bethesda, MD, USA). Fresh tumor samples were stored and frozen within 1 h of surgical resection at − 80 °C.

Adrenal glands were obtained from patients with primary aldosteronism who underwent curative adrenalectomies or from those with renal tumors who underwent radical nephrectomy, including adrenal glands. All adrenal samples were inspected by a skilled pathologist to exclude samples with potential pathological changes. Portions of adrenals were immediately stored and frozen at − 80 °C. The adrenal medullas were manually dissected under stereomicroscope and stored at − 80 °C. Pooled samples of chromaffin tissue were generated by combining isolated adrenal medulla dissected from 10 different individuals each, controlled for sex and surgical indication (nephrectomy vs. adrenalectomy). All participants provided informed consent for inclusion in this study. The study was conducted in accordance with the Declaration of Helsinki, and the protocols were approved by the Ethics Committee of the Faculty Hospital and Medical Faculty of Palacky University in Olomouc (approval no. 13/14) and the Ethics Committee of General University Hospital and First Faculty of Medicine, Prague (approval no. 120/14).

### Mutation screening

Genetic testing for PPGL susceptibility genes was performed for each patient as part of their NIH clinical research evaluation or at their referral institution. Germline and somatic mutations in PPGL susceptibility genes are summarized in Table 1. Eighteen of the 22 patients had germline mutations in PPGL susceptibility genes. In nine patients, *SDHB* mutations were found, five had *VHL* mutations, and four had *RET* mutations. One patient (no. 15) carried a somatic mutation in the *EPAS1/HIF2A* gene and was negative for 19 germline mutations (National Heart, Lung, and Blood Institute [NHLBI] testing panel: *RET, MAX, VHL, SDHA/B/C/D/AF2, TMEM127, NF1, KIF1Bbeta, EGLN1, EGLN2, K-RAS, IDH1, IDH2, FH, MDH2, and HIF2A*). Three patients (Nos. 20, 21, and 22) were negative for mutations in well-known PPGL susceptibility genes. Tumors from two of these patients (Nos. 20 and 22) were concluded to be sporadic based on a negative family history for PPGL and negative genetic testing; the first included eight genes (Genetic PPGL Mayo Testing: *RET, MAX, VHL, SDHB/C/D/AF2*, and *TMEM127*) and the second included 19 genes (NHLBI testing: *RET, MAX, VHL, SDHA/B/C/D/AF2*, *TMEM127, NF1, KIF1Bbeta, EGLN1, EGLN2, K-RAS, IDH1, IDH2, FH, MDH2, and HIF2A*). The tumor from the third patient (No. 21) was also considered sporadic after negative genetic testing for *RET* (Genetic PPGL Mayo Testing) due to clinical suspicion of multiple endocrine neoplasia (MEN), despite having a somatic *CTNBB1* somatic mutation (currently not implicated in hereditary PPGL) somatic mutation. For the remaining three patients (Nos. 10, 13, and 16), genetic testing reports were not available, but their clinical presentation and gene mutations listed and described in the clinical notes were conclusive for mutations attributed to PPGLs. Only two tumor samples (Nos. 15 and 21) were tested for somatic mutations.

**Table 1:** PPGL patient-sample information. PGL–paraganglioma, PHEO–pheochromocytoma

Patient No.	Age at surgery	Sex	Tumor type	PPGL Cluster	Mutated Gene
1	64	F	PGL	1A	*SDHB*
2	39	F	PGL	1A	*SDHB*
3	31	M	PGL	1A	*SDHB*
4	17	M	PGL	1A	*SDHB*
5	34	M	PGL	1A	*SDHB*
6	36	F	PGL	1A	*SDHB*
7	27	M	PGL	1A	*SDHB*
8	56	F	PGL	1A	*SDHB*
9	13	F	PGL	1A	*SDHB*
10	11	M	PHEO	1B	*VHL*
11	44	F	PHEO	1B	*VHL*
12	30	F	PHEO	1B	*VHL*
13	39	M	PGL	1B	*VHL*
14	23	M	PGL	1B	*VHL*
15	34	F	PHEO	1B	*EPAS1*
16	29	M	PHEO	2	*RET*
17	48	F	PHEO	2	*RET*
18	31	M	PHEO	2	*RET*
19	28	F	PHEO	2	*RET*
20	52	F	PHEO	Unassigned	-
21	59	F	PHEO	Unassigned	-
22	61	M	PHEO	Unassigned	-

### Proteomic analyses

#### High pH–trypsin–cyanogen bromide (hpTC) method

hPTC method was applied in the same way as described in [8]. Briefly, approximately 100 mg of tissue pulverized in liquid nitrogen was kept in hypotonic buffer (10 mM NaCl, 2 mM MgCl_2_, 10 mM HEPES, pH 7.4) for 15 min and passed through a 20 G hypodermic needle repeatedly. The homogenate was centrifuged at 500 × *g* 5 min, and the supernatant was treated with 120 Kunitz units of bovine deoxyribonuclease I with 25 mM MgCl_2_ and 5 mM CaCl_2_ for 30 min at 37 °C, then centrifuged at 20,000 × *g*, 4 °C. The resuspended pellet was shaken in 100 mM Na_2_CO_3_ with 1 mM EDTA for 30 min on ice, and this step was repeated once. The pellets were resuspended again in 50 mM NH_4_HCO_3_ with 20 µg trypsin (Promega) and incubated at 37 °C overnight. The suspension was pelleted and washed with Na_2_CO_3_ twice and three times, snap-frozen on dry ice, and thawed between the washes. The pellet was solubilized in 70% trifluoroacetic acid (TFA) with CNBr (20 mg/mL) and incubated in the dark at room temperature overnight. The evaporated material was solubilized in 80% acetonitrile, 10% isopropanol, and 5% formic acid (FA), and diluted 1:10 with 0.5% FA. The sample was desalted delipidated on an OptiTrap column (Optimize Technologies): flushed with 0.5 mL 0.5% FA, then 2.5 mL dichloromethane with 0.5% FA, then 0.5 ml 0.5% FA, and eluted with 80% acetonitrile, 10% isopropanol, 0.5% FA.

#### Solid phase enrichment of N-linked glycopeptides (SPEG)

The method was used as in [9]. Briefly, approximately 100 mg of tissue pulverized in liquid nitrogen was resuspended in a high-salt buffer (2 M NaCl, 1 mM EDTA, 10 mM HEPES-NaOH, pH = 7.4), sonicated, and centrifuged at 20,000 × *g* at 4 °C for 30 min. The pellet was resuspended and washed twice with 100 mM Na_2_CO_3_ and 1 mM EDTA (as in hpTC). This membrane fraction was digested according to [10]: the pellet was solubilized in 5% sodium deoxycholate (SDC) in 100 mM NH_4_HCO_3_, kept for 15 min at room temperature, resuspended, and sonicated. The insoluble debris was removed by centrifugation at 10,000 × *g* for 5 min. The sample was reduced and alkylated (20 mM dithiothreitol, 45 mM iodoacetamide), and the sample was diluted to final 1% SDC and 50 mM NH_4_HCO_3_. Trypsin (Promega) was added at a 1:50 trypsin:protein ratio and the sample was kept at 37 °C overnight. After digestion, TFA was added to pH < 3, and SDC was removed by phase transfer (ethyl acetate added at 1:1 vol, 1 min shaking, and centrifugation at 15,000 × *g* for 2 min) which was 4 times repeated. The sample was desalted using an OptiTrap column (Optimize Technologies), according to the manufacturer’s instructions.

The desalted peptides were solubilized in 500 µL of Affi-Gel Hz Coupling Buffer (Bio-Rad), oxidized with 10 mM NaIO_4_ for 1 h in the dark, and quenched for 10 min by the addition of 20 mM Na_2_S_2_O_3_. Affi-Gel Hz Hydrazide Gel beads (Bio-Rad) were washed ddH_2_O and twice in Coupling Buffer, the sample was added, and the beads were rotated overnight. The beads were then washed thoroughly (1.5 M NaCl, 80% acetonitrile, and 50 mM NH_4_HCO_3_, 1 ml each) and 3 units of peptide N-glycosidase F (PNGase F, Roche) were added to 25 µL of 50 mM NH_4_HCO_3_. The beads were rotated at 37 °C overnight. The supernatant and additional washes (2 × 50 µL of 50 mM NH_4_HCO_3_, 40 µL of 0.5 M NaCl and 40 µL of 80% acetonitrile) were collected, and the sample was desalted using OptiTrap column again.

#### N-glycopeptide enrichment with filter-aided sample preparation (N-glyco-FASP)

The initial protein digest of the membrane fraction was prepared in the same manner as for SPEG. The desalted peptides were solubilized in binding buffer (0.5 M NaCl, 1 mM MnCl_2_, 1 mM CaCl_2_, 20 mM tris-HCl, pH = 7.6) and 100 µg of wheat germ agglutinin, 100 µg of concanavalin A and 80 of µg Ricinus communis agglutinin I (RCA120) were added. The mixture was kept for 1 h on a rocker on a 10 kDa ultrafilter spin column (Microcon Ultracel-10 Membrane, 10 kDa), washed four times with 200 µL of binding buffer, twice with 50 mM NH_4_HCO_3_ and kept in 40 µl of 50 mM NH_4_HCO_3_ with 2 units of PNGase F at 37 °C overnight. The filter flow-through was collected by centrifugation together with additional washes (2 × 50 µL of 50 mM NH_4_HCO_3_ and 40 µL of 0.5 M NaCl). The samples were desalted using an OptiTrap column.

#### SDC-trypsin

Approximately 10 mg of tissue was pulverized in liquid nitrogen and digested according to [10]. The material was resuspended, sonicated in 5% SDC in 100 mM NH_4_HCO_3_ and digested the same way as described above (see SPEG).

### nLC-MS/MS analysis

The peptide samples were separated on a reversed-phase nano column (EASY-Spray column, 50 cm × 75 μm ID, PepMap C18, 2 μm particles, 100 Å pore size). Mobile phase buffer A was composed of water, 2% acetonitrile, and 0.1% formic acid. Mobile phase B was composed of 80% acetonitrile, 0.1% formic acid. Samples were loaded onto a trap column (Acclaim PepMap300, C18, 5 μm, 300 Å Wide Pore, 300 μm × 5 mm, 5 Cartridges) for 4 min at 15 µl/min, with a loading solution composed of water, 2% acetonitrile, and 0.1% TFA. After 4 min, the valve was switched, and mobile phase B was increased from 4 to 35% B in 120 min at 300 nl/min, followed by a wash with 75% B 5 min at 400 nl/min, and then 4% B for 5 min until the end of the run (for all samples except hpTC). For the hpTC samples, the mobile phase B was increased from 4 to 50% B in 120 min at 300 nl/min, followed by a wash with 75% B 5 min at 400 nl/min, and then 4% B for 5 min until the end of the run. Eluting peptide cations were converted to gas-phase ions by electrospray ionization and analyzed using a Thermo Orbitrap Fusion (Q-OT-qIT, Thermo). Survey scans of peptide precursors from 350 to 1400 m/z were performed at 120 K resolution (200 m/z) with a 5 × 105 ion count target. Tandem MS was performed by isolation at 1.5 Th with quadrupole, HCD fragmentation with a normalized collision energy of 30, and rapid-scan MS analysis in the ion trap. The MS2 ion count target was set to 104, and the maximum injection time was 35 ms. Only precursors with charge states 2–6 were sampled for MS2. The dynamic exclusion duration was set to 45 s with a 10 ppm tolerance around the selected precursor and its isotopes. The monoisotopic precursor selection was performed. The instrument was run in the top-speed mode with 2 s cycles [11].

### Data processing

Raw mass spectrometry data files were analyzed and quantified using MaxQuant v1.6.0.7. The data were searched against the human subset of the Swiss-Prot database (20,395 sequences, May 2021) using different settings for each method. Trypsin/P with 2 max missed cleavages was present in all methods; in hpTC, CNBr (cleavage C-terminal to methionine) with 2 max missed cleavage sites was set. For all methods, N-terminal protein acetylation and methionine oxidation were used as variable modifications. In glyco-capture (SPEG and N-glyco-FASP, searched together as two fractions for each sample), asparagine deamidation was set, and in hpTC, methionine (C-terminal to any peptide) substitutions to homoserine lactone (Met − 48.003) and homoserine (Met − 29.993) were set. Carbamidomethylation of cysteine was set as a fixed modification in all methods except hpTC. The false discovery rate (FDR) was set to 1% for both, proteins and PSM.

Using these parameters, label-free quantification (LFQ) searches were performed, hpTC, glyco-capture, and trypsin separately, for all sets of tumor samples (cluster 1, cluster 2, and unassigned) with the control samples. In the resulting datasets, the LFQ intensity values were further normalized such that the sum of all intensities was equivalent to the average sum of the original intensities in the dataset. The datasets were further processed using Perseus v1.6.2.3. Protein groups identified based on at least two unique peptides present in at least 80% of either tumor samples or control tissue were kept. 4-fold upregulated protein levels (p < 0.05) were considered significant.

To identify integral membrane proteins, deepTMHMM agorithm [12] was used to predict the transmembrane segments. The cell surface proteins were identified using the GO annotation GO:0005886 “Plasma membrane” via the function “Retrieve/ID mapping“ on UniProt (https://www.uniprot.org/).

Unsupervised hierarchical clustering was performed using Perseus v1.6.2.3, based on an LFQ search containing all the “SDC-trypsin” samples. The data were processed in the same way as above, missing values were imputed from normal distribution, data were subjected to ANOVA, significant values were normalized by Z-score, and the hierarchical clustering function was executed with its default parameters. The mixOmics 6.16.3 R package was used for supervised discrimination analysis (sPLS-DA).

### SDS-PAGE and western blotting

The samples for SDS-PAGE and western blotting were prepared the same way as samples for SDC-trypsin prior to reduction, alkylation, and digestion, in 5% SDC and 100 mM NH_4_HCO_3_. The protein concentration was determined using a bicinchoninic assay. The samples were mixed with sample buffer (final 2% SDS, 10% glycerol, and 5% 2-mercaptoethanol). The samples were separated on 10% gels with 5% stacking front, containing 29:1 acrylamide:bis solution (Bio-Rad) in Mini-Protean TetraCell module (Bio-Rad) in Tris/Glycine/SDS buffer (Bio-Rad). The electrophoreses were transferred to Trans-Blot Turbo Mini 0.2 μm PVDF membranes in the Trans-Blot Turbo Transfer System (Bio-Rad), washed in PBS with 0.1% Tween 20 (PBST), blocked in 5% (w/v) skimmed milk in PBST for 30 min, washed 3 times/5 min in PBST, sealed in small volume with primary antibody, and incubated for 15 min at room temperature and at 4 °C overnight. The membranes were washed 3 times/10 minutes in PBST, incubated with horseradish peroxidase-conjugated secondary antibody for 30 min, washed 3 times/10 minutes in PBST, and once in PBS. The membranes were incubated with KPL LumiGLO Chemiluminescent Substrate (SeraCare), and chemiluminescence was detected using the ChemiDoc MP System (Bio-Rad). The detected bands were quantified using ImageLab 6.0.1 (Bio-Rad) and normalized against total lane optical densities of Coomassie Brilliant Blue stained gel run in parallel with the gel used for western blotting.

The following antibodies were used at corresponding dilutions: anti-ACE, 1:100 (sc-23,908; Santa Cruz); anti-ANO1, 1:1000 (MA5-16358, Invitrogen); anti-ARG2, 1:500 (ab137069, Abcam); anti-ATP8A1 1:1000 (21,565-I-AP, Proteintech), anti-ATX 1:100 (sc-374,222, Santa Cruz), anti-CD39, 1:1250 (ab108248, Abcam); anti-CD146, 1:200 (ab24577, Abcam); anti-CTR1, 1:500 (NBP1-91447, Novus Biologicals); anti-L1CAM, 1:100 (sc-53,386, Santa Cruz); and anti-SHMT2, 1:1000 (HPA020543, Merck). Secondary antibodies were as follows: anti-mouse-HRP, 1:10 000 (115-035-003, Jackson) and anti-rabbit-HRP, 1:10 000 (711-035-152, Jackson).

## Results

This study focused on cluster 1 PPGLs, which are characterized by a high risk of metastasis, multiplicity, or recurrence. The set of 22 analyzed tumors comprised of cluster 1 A PPGLs (n = 9) with *SDHB* pathogenic variants, cluster 1B (n = 6) with *VHL* and *EPAS1* pathogenic variants, cluster 2 PPGLs (n = 4) with *RET* pathogenic variants and 3 “unassigned” PPGLs (tumors without pathogenic variants in the known PPGL-associated genes). For more patient information, please see Table 1. Five samples of microdissected and pooled human normal adrenal medulla (NAM) represented the control chromaffin tissue in our study (each pooled sample represented the adrenal medulla from 10 individuals).

### Standard LFQ-proteomic analysis of total lysates

**Fig. 1:**
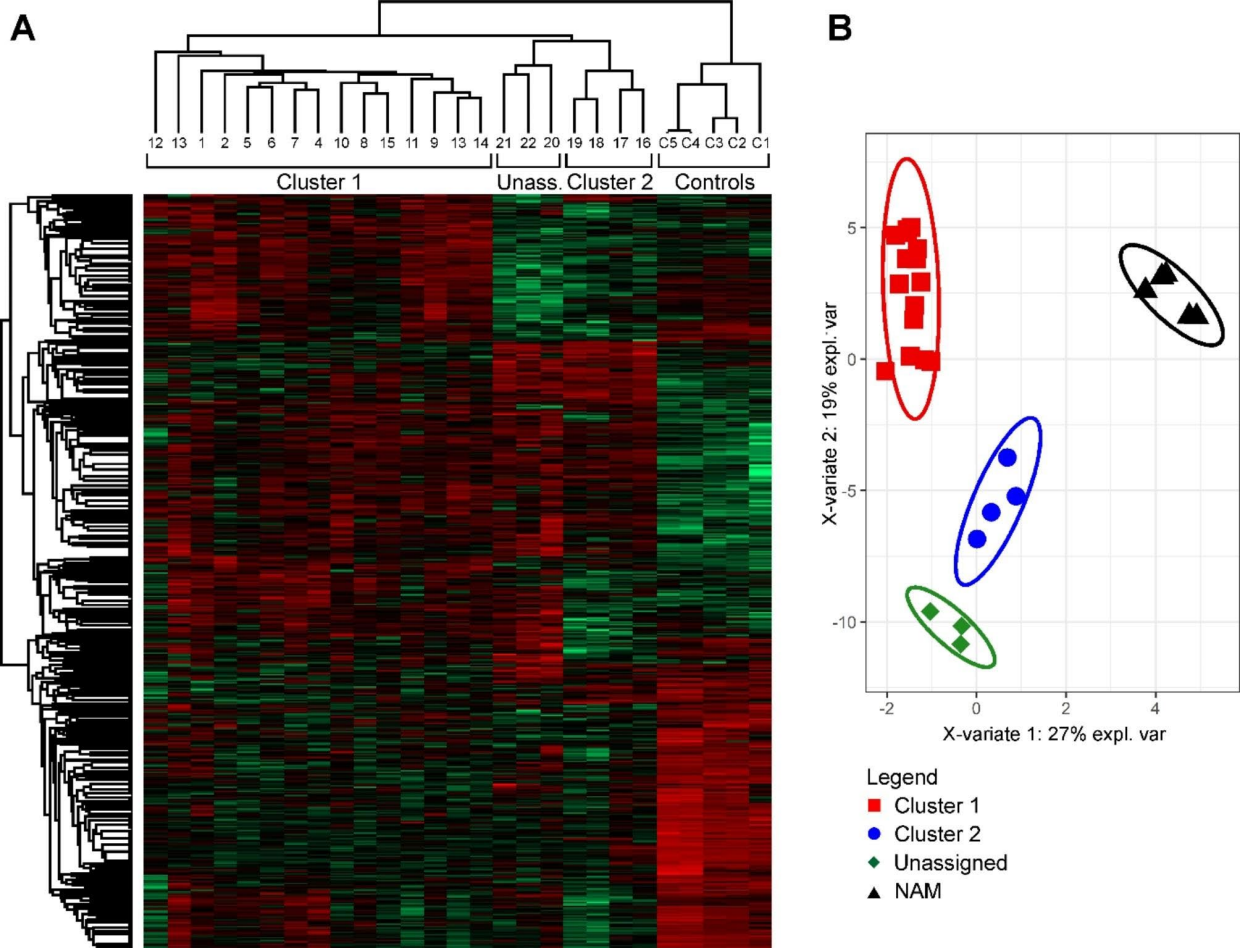
Analysis of protein expression data from the standard proteomic analysis of total tumor and control tissue lysates. Both data analyses confirmed the robustness of the data and demonstrated the biological differences among the tumor clusters via clear separation of normal adrenal medulla (NAM), cluster 1, cluster 2, and unassigned pheochromocytomas and paragangliomas (PPGLs). (**A**) Unsupervised hierarchical clustering of the protein expression data. (**B**) Supervised sparse partial least square discrimination analysis (sPLS-DA) of the expression data

Standard LFQ LC-MS/MS of trypsin-digested total lysates of the 22 PPGL samples and 5 control NAM samples identified and quantified almost 3000 proteins with at least two unique peptides (FDR for protein and peptides 0.01). Unsupervised hierarchical clustering (Fig. 1A) and sPLS-DA clearly separated NAM, cluster 1, cluster 2, and unassigned PPGL samples (Fig. 1B). This confirms the molecular basis of the original cluster assignment and correct tumor classification, and reflects the reproducibility of our analyses.

**Fig. 2:**
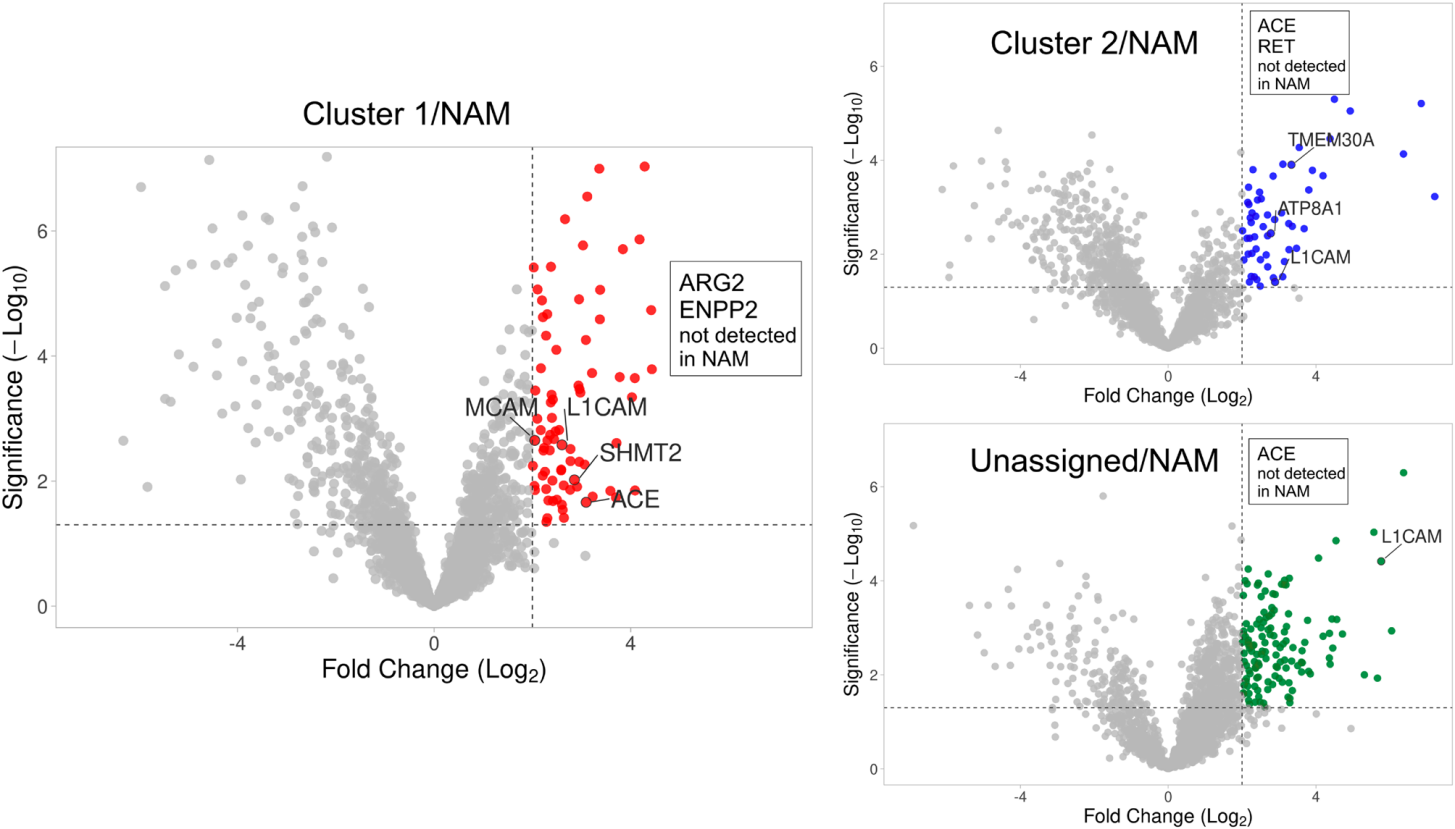
Standard proteomic analysis. Proteins differentially upregulated in PPGLs relative to control tissues (NAM). Volcano plots (protein expression fold-change plotted against the statistical significance) separately for each PPGL group (cluster 1, cluster 2, and unassigned PPGLs). Colored spots indicate the proteins upregulated (threshold 4-fold, p-val 0.05) in each tumor group than in the control tissues (NAM). Gene names for the corresponding proteins are shown for clarity, and only for the proteins discussed further. Upregulated proteins detected in tumors but absent in NAM could not be plotted (due to the missing fold-change and significance values), those of interest are therefore listed in the inset boxes. MCAM, melanoma cell adhesion molecule (CD146); L1CAM, neural cell adhesion molecule L1 (CD171); SHMT2, serine hydroxymethyl transferase 2; ACE, angiotensin-converting enzyme; ARG2, arginase 2; ENPP2, ectonucleotide pyrophosphatase/phosphodiesterase family member 2 (autotaxin); ATP8A1, phospholipid-transporting ATPase IA; TMEM30A, cell cycle control protein 50 A

As the goal of our study was to identify new potential druggable targets or tumor imaging markers, we considered only massively upregulated proteins (at least 4-fold relative to NAM, p < 0.05). We identified 125, 105, and 174 markedly upregulated proteins in cluster 1, cluster 2, and unassigned tumors, respectively (Fig. 2), compared to NAM. The lists of upregulated proteins also include “ON/OFF” proteins, i.e. proteins detected in all tumor samples in a given group (cluster 1, cluster 2, or unassigned) but absent in all of the 5 NAM samples. For such proteins, the fold-change and p-value could not be calculated due to missing values in the control samples. Therefore, these proteins are not displayed in volcano plots. The list of all differentially expressed tumor proteins identified in the standard proteomic analysis is shown in Additional File 1.

**Fig. 3:**
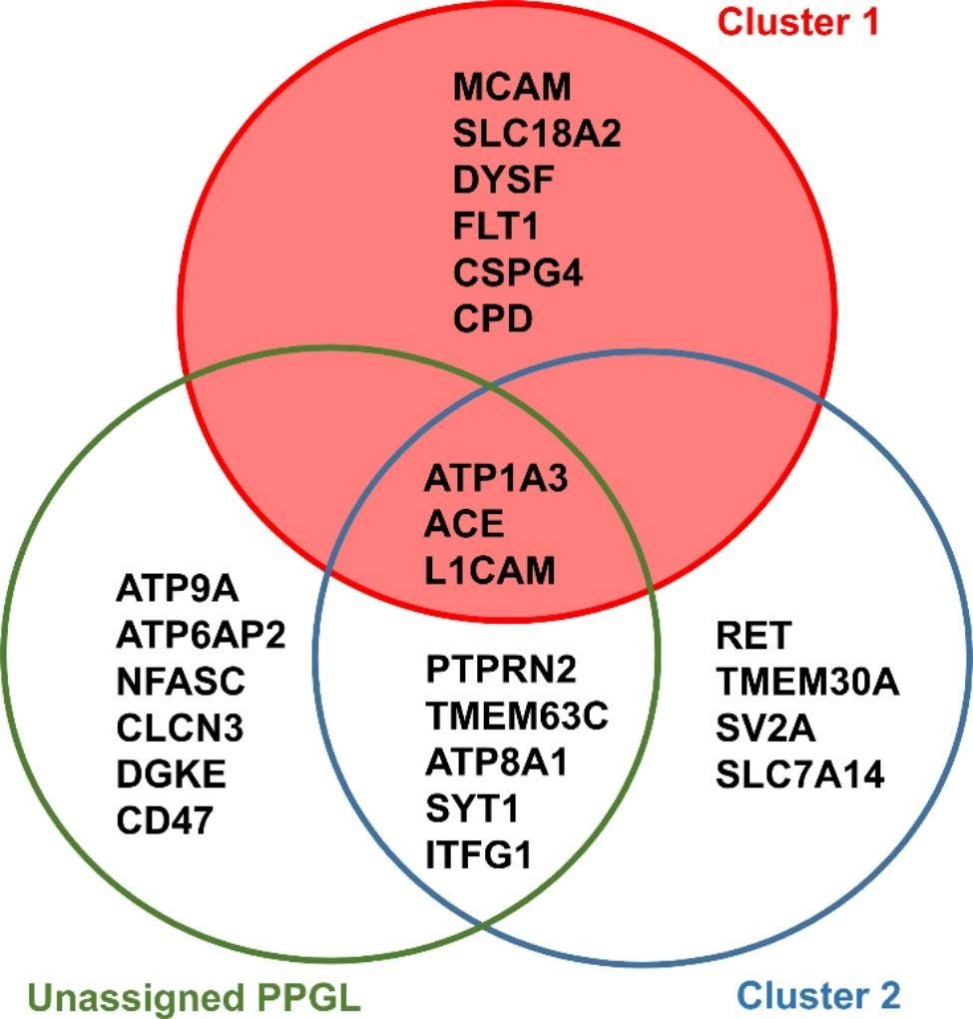
Differentially upregulated cell-surface integral membrane proteins (IMPs) identified via standard total lysate proteomic analysis in cluster 1, cluster 2, and unassigned PPGLs. Primary gene names are used instead of protein names. Differentially expressed proteins were filtered for presence of predicted transmembrane domains and their cell surface localization

To search for potential druggable targets, we first reduced the list of identified upregulated proteins to contain only integral membrane proteins (IMPs). We found 20, 21, and 26 upregulated IMPs in cluster 1, cluster 2, and unassigned PPGL, respectively (based on the deepTMHMM prediction [12]). Of the upregulated IMPs, we further considered only those localized on the cell surface (i.e. IMPs with G.O annotation “plasma membrane” (GO:0005886) assigned according to UniProt). Nine upregulated cell-surface IMPs were found in cluster 1: MCAM (CD146), FLT1 (VGFR1), CDP (carboxypeptidase D), CSPG4 (chondroitin sulfate proteoglycan 4), DYSF (dysferlin), SLC18A2 (VMAT2), L1CAM (CD171), ATP1A3 (sodium/potassium-transporting ATPase subunit alpha-3), and angiotensin-converting enzyme (ACE). The last three proteins were also upregulated in cluster 2 and unassigned PPGL relative to NAM. Additional cell-surface IMPs were identified as upregulated exclusively only in cluster 2 (4 IMPs, including the cluster 2 “landmark” protooncogene RET) or unassigned PPGL (6 IMPs) or shared by these two tumor groups (5 IMPs) (Fig. 3).

In total, only 24 markedly upregulated cell-surface IMPs were identified in all tumor groups combined, based on the standard “total lysate” analysis. IMPs are coded roughly by 25% percent of human genes [13]. However, it is well known that because of their low expression, low solubility, and lack of trypsin cleavage sites in transmembrane segments, IMPs are generally under-represented in standard proteomic analyses, and specific strategies must be applied to increase IMP coverage [14, 15].

### Application of multi-pronged pitchfork strategy

**Fig. 4:**
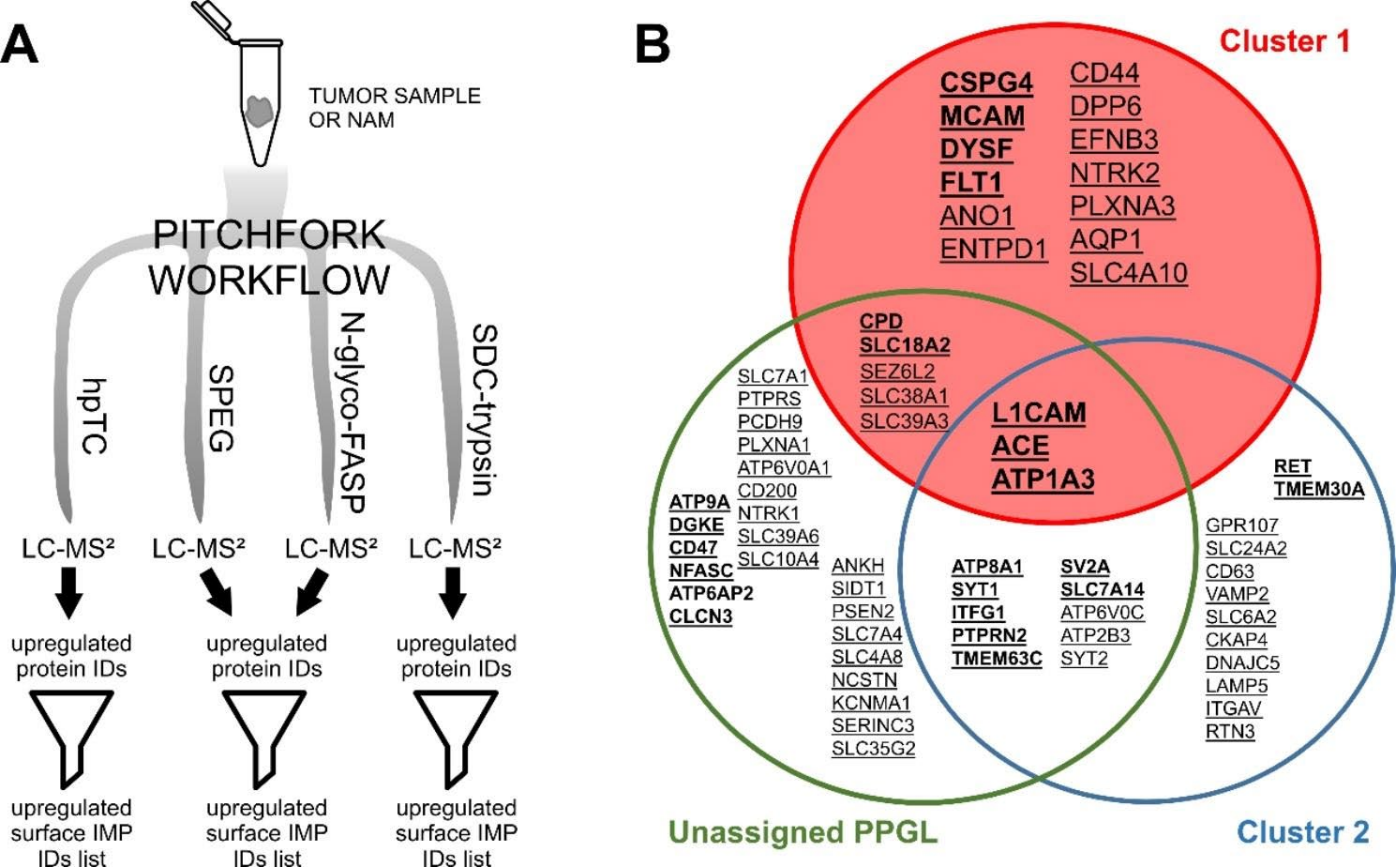
Multi-pronged Pitchfork strategy (**A**) Pitchfork strategy workflow: all tumor and control (NAM) samples were analyzed using four parallel methods and different ways of sample processing with separate liquid chromatography-tandem mass spectrometry (LC-MS/MS) analyses. (1) SDC-trypsin: standard proteomic analysis of total sample lysate, (2) N-glyco-FASP: glycopeptide enrichment using lectins, (3) SPEG: glycopeptide enrichment using hydrazide chemistry, and (4) hPTC: enrichment of hydrophobic membrane-embedded segments of IMPs. MS data were searched and proteins identified and quantified (LFQ) for each of the method separately, with the exception of the two glycopeptide “prongs,” where the MS data from both glycocapture methods were searched together. Identified upregulated proteins were filtered for presence of predicted transmembrane segment (TMHMM) and the resulting list of upregulated IMPs was further filtered for cell-surface localization. (**B**) Upregulated cell-surface IMPs identified in the 22 analyzed PPGLs using the complete Pitchfork workflow (corresponding primary gene names are shown). Proteins identified via standard proteomic analysis are shown in bold letters, proteins identified using glycocapture or hPTC are underlined. Proteins identified using both, total cell lysate analysis and membrane-aimed methods, are indicated by **underlined bold letters**

We recently presented a multi-pronged Pitchfork strategy that extracts maximum information on the membrane proteome by targeting specific features of IMPs. This strategy complements standard total lysate proteomic analysis with parallel LC-MS/MS analyses of enriched glycopeptides and hydrophobic transmembrane segments [9] (Fig. 4A).

### N-glyco-FASP and SPEG

Prevalent N-glycosylation of IMPs on their extracellular or luminal segments enables the enrichment of intact IMPs or their glycosylated peptides via glycan-affinity to lectins (N-glyco-FASP) or via hydrazide chemistry and solid-phase extraction of N-linked glycopeptides (SPEG) [6, 7]. In the current study, we used both enrichment methods in parallel and combined the MS data obtained from the two glycopeptide-enrichment methods into one search to maximize the number of peptides per protein for the LFQ analysis and to increase the number of proteins confidently identified. Combined glycocapture-based analysis showed that 17, 20, and 18 markedly upregulated IMPs (at least 4-fold compared to NAM) in cluster 1, cluster 2, and unassigned PPGLs, respectively. Of these, 12, 13, and 15 were cell surface proteins. Among those, ENTPD1 (CD39), DDP6, and CD44 were identified in cluster 1, andCD63 was found in cluster 2 (Fig. 4B). The list of all differentially expressed proteins, IMPs and cell surface IMPs identified by glyco-capture is shown in Additional File 1. Volcano plots are available as Additional File 2.

### hPTC analysis

Hydrophobic transmembrane segments, normally inaccessible to standard proteomic analysis, represent another specific feature of IMPs that can be used to enrich and identify IMPs. Upon tryptic digestion of all non-membrane proteins and extracellular segments of IMPs, hydrophobic membrane-protected segments can be solubilized, cleved chemically at methionines with CNBr, and subjected to LC-MS/MS, as originally demonstrated by Blackler et al. [16]. We previously modified and streamlined the approach and presented it under the hpTC acronym (high pH-trypsin-CNBr) [8]. Here, hpTC analysis was applied to all PPGL and control samples, and 9, 9, and 34 upregulated IMPs were identified in cluster 1, cluster 2, and unassigned PPGLs, respectively. Six, 3, and 20 of these are cell surface proteins, as exemplified by anoctamin-1 (ANO1) in cluster 1) and high-affinity cationic amino acid transporter (SLC7A1) in unassigned PPGLs. The list of all differentially expressed proteins, IMPs and cell surface IMPs identified by hPTC is shown in Additional File 1. Volcano plots for hPTC are available as Additional File 2.

The hPTC, glyco-capture and the standard trypsin-based proteomic analysis all aim at different properties of IMPs, and provide protein identification with different peptide sets. While the standard analysis provides mostly hydrophilic peptides, glyco-capture methods enrich N-glycosylated peptides while hPTC provides hydrophobic membrane-embedded segments [6, 7]. Here, this complementarity can be demonstrated on examples of differentially expressed IMPs, namely anoctamin 1 (ANO1), high-affinity cationic amino acid transporter 1 (SLC7A1) and CD39 (ENTPD1). Their topology prediction with mapped identified peptides are shown in Additional File 3.

Together, the standard proteomic analysis, two glycocapture methods, and hpTC analysis representing the whole Pitchfork strategy provided a list of 67 cell surface IMPs markedly and significantly upregulated in PPGLs relative to NAM (Fig. 4B).

### Confirmation

**Fig. 5:**
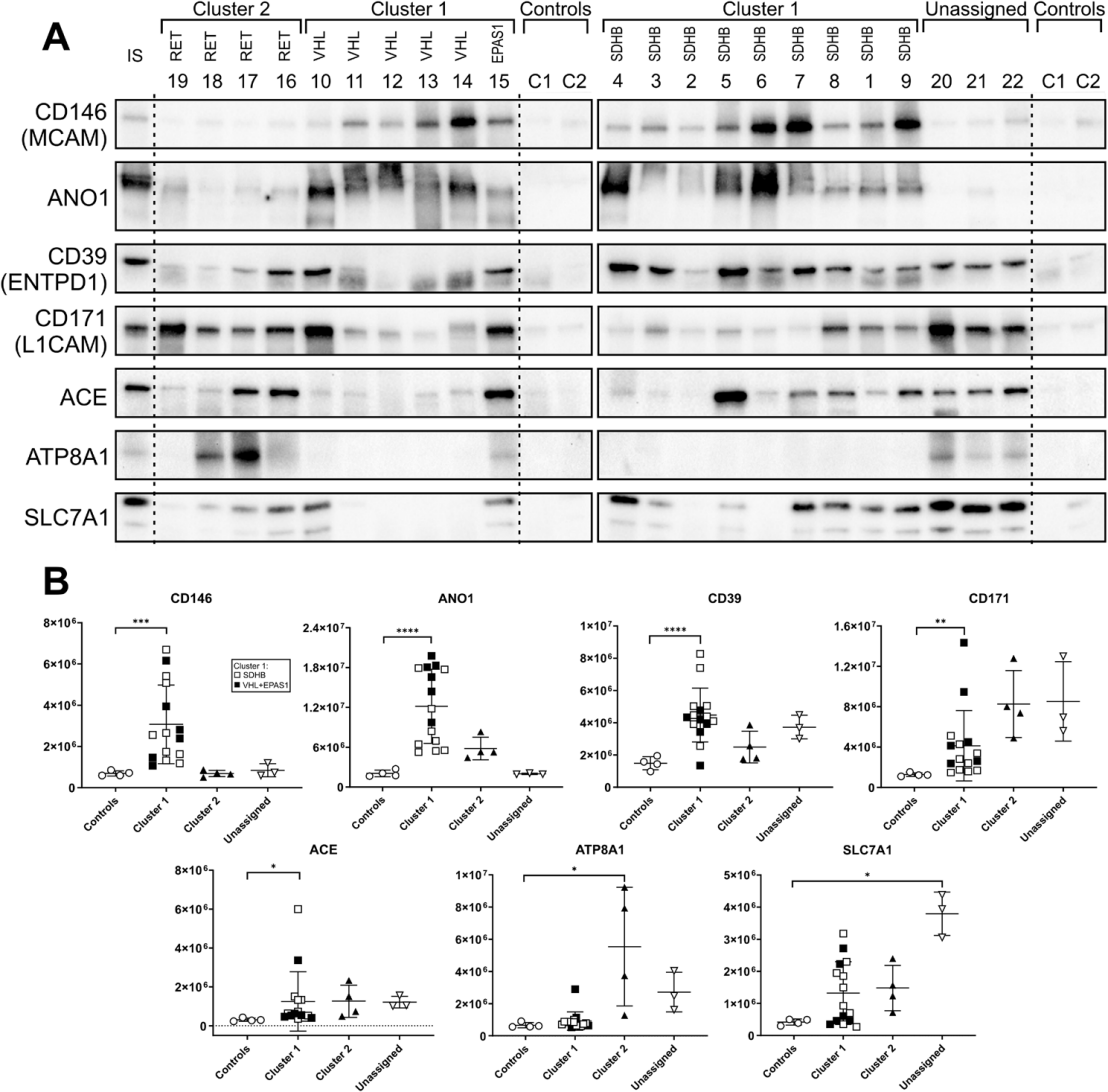
Antibody-based confirmation of the selected upregulated cell-surface IMPs. (**A**) Western blots of 22 tumor samples and 2 controls (NAM pools). (**B**) Results of densitometric analyses of the blots. To normalize the signal intensity between the 2 blots, either sample 22 or sample 4 (in case of CD146 and ANO1) are present on both membranes and used as an internal standard. Due to the heterogeneity of tumors, standard loading “housekeeping“ proteins, such as glyceraldehyde-3-phosphate (GAPDH), tubulin, and actin, could not be used, as their antibody-detected levels varied significantly among the individual samples. Therefore, we used a Coomassie Brilliant Blue-stained gel as a loading control. ANO1, anocatamin 1 (TMEM16A); ACE, angiotensin-converting enzyme; ATP8A1, phospholipid-transporting ATPase IA; SLC7A1, high-affinity cationic amino acid transporter 1

We selected five high-priority proteins from the list of cell surface IMPs upregulated in cluster 1 PPGLs for verification with specific antibodies. To identify new drug targets or targets for tumor-imaging tracers, we prioritized proteins with previously established direct roles in cancer growth. As the most perspective in the cluster 1 we selected CD146 (MCAM), Anocatmin-1 (ANO1), CD39 (ENTPD1), CD171 (L1CAM) and angiotensin-converting enzyme (ACE). Additionally, to verify the robustness of the proteomic data in low-risk cluster 2 and unassigned tumors (both groups were represented by a lower number of tumor samples), we verified the upregulation of a prospective cell surface drug targeting ATP8A1 in cluster 2 PPGLs and SLC7A1 in unassigned PPGLs. The immunoblots confirmed the results of the proteomic analyses, demonstrating marked (at least 3-fold) and statistically significant upregulation of all seven proteins in one or more PPGL groups. (Fig. 5)

### Non-membrane druggable proteins

In addition to cell surface IMPs, potential drug targets can also be found in soluble druggable enzymes. Although primarily membrane-oriented, the Pitchfork strategy also provides information on soluble cellular proteins, as it includes a standard “total lysate” analysis. Here, we provide quantitative information on approximately 3000 proteins across all tumor samples. KEGG pathway enrichment analysis of proteins identified as significantly upregulated in cluster 1 suggested potential changes in “biosynthesis of amino acids” (hsa1230, adjusted p-value = 0.0008). We identified several druggable molecules as putative therapeutic targets among the upregulated enzymes that participate in these metabolic pathways. Among the most promising are two mitochondrial enzymes, **serine hydroxymethyl transferase 2 (SHMT2) and arginase 2 (ARG2)** responsible for the conversion of mitochondrial serine to glycine and arginine to ornithine, respectively. Experimental low-molecular-weight inhibitors are available for both enzymes, and both proteins were previously shown to be directly related to cancer progression in studies exploring their inactivation or inhibition in cancer cells. Additionally, we prioritized secreted lysophosphatidase **autotaxin (ENPP2)** as another putative drug target with already established direct roles in cancer and LMW inhibitors.

**Fig. 6:**
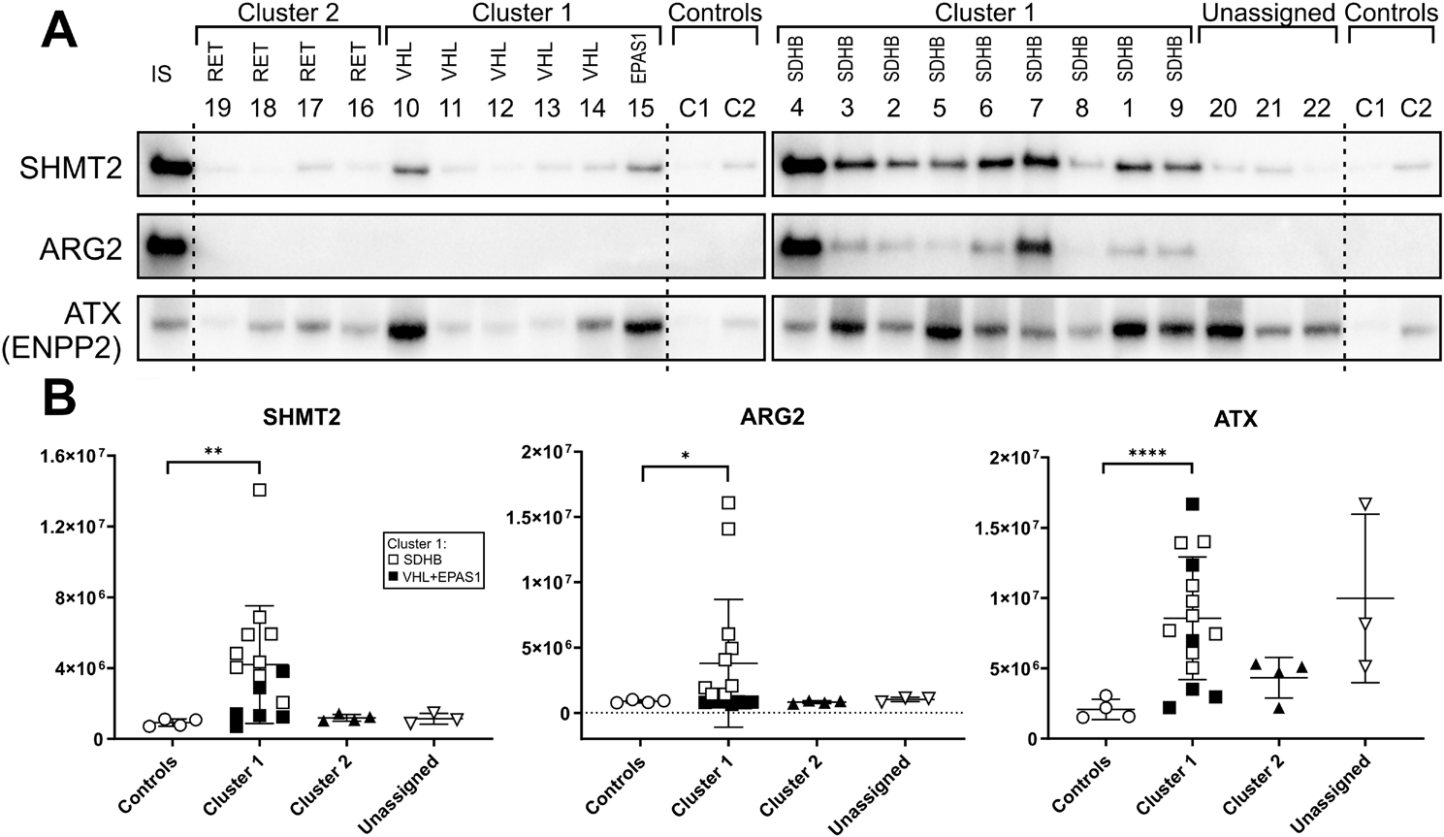
Antibody-based confirmation of selected druggable enzymes markedly upregulated in cluster 1. (**A**) Western blots of 22 tumor samples and 2 controls (NAM pools). (**B**) Results of densitometric analyses of the blots. To normalize the signal intensity between the two blots, sample 4 was present on both membranes and used as an internal standard. Due to the heterogeneity of tumors, standard loading “housekeeping“ proteins, such as GAPDH, tubulin, actin, could not be used, as their antibody-detected levels varied significantly among the samples. Therefore, we used a Coomassie Brilliant Blue-stained gel as the loading control. SHMT2, serine hydroxymethyl transferase 2; ARG2, arginase 2 (mitochondrial); ATX, autotaxian alias ENPP2, ectonucleotide pyrophosphatase/phosphodiesterase family member 2

Using specific antibodies, we confirmed a marked (> 4-fold) upregulation of these three druggable enzymes compared to NAM (Fig. 6). SHMT 2 was confirmed to be significantly upregulated in cluster 1 tumors, most markedly in tumors with *SDHB* pathogenic variants. Similarly, mitochondrial arginase 2 was markedly upregulated in *SDHB* pathogenic variant cluster 1 PPGLs, with virtually no signal detected in controls or other tumors, including *VHL* and *EPAS1* pathogenic variant cluster 1 PPGLs. The *SDHB*-specific upregulation of both enzymes may represent an adaptive compensation for the SDHB defect affecting both TCA and/or Complex II of the ETC. Autotaxin was confirmed to be upregulated in cluster 1 and unassigned PPGLs.

## Discussion

Finding new druggable targets and/or targets for ligands enabling PET/CT tumor imaging of PPGLs, with an emphasis on high-risk cluster 1 PPGLs, was the ultimate motivation behind the current study. Plasma membrane proteins overexpressed by tumors are excellent targets applicable to both tumor imaging and/or therapy because of their accessibility from extracellular space and their functions, which are often critical to cancer survival, proliferation, or migration. IMPs are, however, notoriously under-represented in standard proteomic analyses because of their hydropathy, lack of trypsin cleavage sites in hydrophobic segments, and generally low expression. This requires special enrichment and analytical techniques. The recently introduced Pitchfork strategy [9] combines a standard trypsin-based “total lysate” proteomic analysis with two different methods of glycocapture and enrichment of hydrophobic transmembrane peptides. Quadruple analysis of each sample ensured a high membrane proteome coverage. This strategy has already been shown to be effective in a small pilot experiment preceding the current project, where we identified the upregulated cell-surface protein PSMA as a potential theranostic target in PSMA in cluster 1 PPGLs [17].

In the current study, the application of the Pitchfork strategy followed by filtering for cell surface localization resulted in the identification of 21 markedly upregulated (> 4-fold) plasma membrane IMPs in cluster 1 PPGL in the current study. Of these, we prioritized five proteins with pre-established direct roles in cancer, and verified their increased expression using specific antibodies. Significant upregulation in cluster 1 PPGLs was confirmed for all five cell-surface IMPs.

**CD146** is upregulated in cluster 1 PPGLs (MCAM, Cell surface glycoprotein MUC18, Melanoma cell adhesion molecule, MCAM), is a membrane glycoprotein that contains five immunoglobulin (Ig) superfamily related extracellular domains, a transmembrane region, and a short cytoplasmic region. CD146 is involved in cell adhesion, signaling, and regulation of endothelial permeability. Increased expression of CD146 has been reported in several human cancers, and its expression is associated with increased tumor growth and invasiveness in human patients and animal cancer models [18–20]. In addition to cell surface CD146, soluble sCD146 is released by ectodomain shedding of membrane CD146, which enhances angiogenesis and metastasis [21–23]. The direct effect of CD146 on tumor growth has been demonstrated in several studies using genetic inactivation or inhibition with anti-CD146 blocking antibodies in breast cancer [20], neuroblastoma [24] and melanoma [22, 23, 25].

**Anoctamin-1 (ANO1, DOG-1, TMEM16A)** is a calcium-activated chloride channel in the plasma membrane. Anoctamin-1 is upregulated in many tumors, including prostate, breast, colorectal, and lung cancers (reviewed in [26, 27]). Its role and regulation seem to be tissue-dependent [26] as inhibition of ANO1 was shown to suppress proliferation and/or migration and metastatic spread in various cancer cells and in mouse xenograft models in several studies, while having no or opposite effect in some other models [26, 27]. Although the exact role of anoctamin-1 in cancer is unknown, it participates in cancer proliferation and migration by modulating the activity of the MAPK, CAMKII, and EGFR signaling pathways [28, 29]. The NIH-approved anti-asthma drug zafirlukast was shown to be a selective and effective anoctamin-1 inhibitor that suppressed lung cancer growth and progression in cell culture and mouse models [30].

**CD39 (ENTPD1)** is a cell-surface IMP with an ecto-nucleotidase function, which hydrolyzes extracellular (immunogenic) eATP to eAMP, which is consequently dephosphorylated via CD73 to (immunosuppressive) adenosine. High expression of CD39 protein has been shown in a multitude of human tumors, including thyroid, pancreatic, lung, prostate [31], and ovarian cancer [32]. CD39 upregulation can be observed in some cancer cells, but mostly in tumor microenvironments, such as fibroblasts, myeloid cells, endothelial cells, and T-cells [33]. The overexpression of CD39 in the tumor microenvironment thus contribute to immunosuppressive character of the milieu. Anti CD39 antibodies and LMW inhibitors can decrease extracellular ATPase activity, increase anti-tumor inflammatory response and reduce tumor growth, as demonstrated in several animal models [34–36]. Efforts to block the CD39/CD73 activity in combination with checkpoint blockade offered highly promising anti-tumor results [35, 36]. Several clinical trials are currently under way to evaluate CD39 monoclonal antibodies as monotherapy or in combination with other agents in cancer patients (clinicaltrials.gov).

Hypoxia, oxidative stress and proinflammatory mediators have been shown to stimulate CD39 expression [37]. Whether the marked upregulation of CD39 seen observed in cluster 1 PPGL can be attributed to the pseudohypoxic character of the cluster 1 tumors or to their actual hypoxic milieu, remains to be determined as well as the particular cell type responsible for overexpression, remains to be determined.

**CD171 (Neural cell adhesion molecule L1, L1CAM)** upregulated in all tested PPGL groups, is a cell surface integral membrane glycoprotein that is essential for normal neural development and regeneration. The expression of CD171 positively correlates with tumor progression and metastasis in different cancer types, including glioma, melanoma, and ovarian cancer (reviewed in [38]). This correlation seems to be related to the ability of CD171 to enhance cancer cell proliferation, invasion, and metastatic dissemination [39, 40]. The role(s) of CD171 in cancer progression seem to be direct, as demonstrated by reduced tumor growth in multiple gene-silencing experiments or with anti CD171 antibodies [38]. A clinical trial of CD171-specific CAR-T cells in patients with neuroblastoma (NCT02311621) is currently underway.

Similar to CD146, soluble sCD171 (sL1CAM) is released into the extracellular space by ectodomain shedding and can be detected in the blood. Increased levels of sCD171 have been detected in the plasma of cancer patients and correlated with disease progression or prognosis in patients with gastrointestinal stromal tumors [41], breast cancer [42], and glioblastoma [43], suggesting the potential of CD171 as a noninvasive circulating tumor biomarker.

**ACE (angiotensin-converting enzyme)** is a well-known component of the renin-angiotensin system (RAS) and a target of approved antihypertensives, such as Captopril and Enalapril. ACE is expressed in several normal and cancerous tissues. It catalyzes the conversion of angiotensin I to angiotensin II, which then plays a pivotal role in regulating blood pressure and vasoconstriction, as well as in inflammation and angiogenesis via nuclear factor-κB (NF-κB) signaling. Various ACE inhibitors have been shown to suppress tumor growth and/or blood vessel formation in mouse models of squamous cell cancer [44], hepatocellular carcinoma [45], pancreatic cancer [46] and renal cell carcinoma [47]. Several retrospective studies have shown the positive effect of ACE inhibitors on the outcome of some antineoplastic therapies (reviewed in [48]). The tumor-promoting actions of RAS have been reviewed elsewhere [48, 49], however, the complex local and systemic roles of RAS in tumor growth, vascularization, and anti-tumor immune responses remain elusive.

The five aforementioned cell-surface proteins identified and confirmed as upregulated in cluster 1 PPGLs were all previously shown to play a direct role in cancer growth, as their inhibition suppressed tumor growth in experimental studies. For all five putative drug targets, LMW inhibitors, blocking antibodies, and other experimental strategies exist to validate their therapeutic potential in future studies.

Although our primary focus was cluster 1, the set of analyzed samples also included cluster 2 tumors and PPGL of patients with no known mutations, albeit represented by only four and three samples, respectively. Of the upregulated cell-surface IMPs identified in these two groups, we verified the increased expression of ATP8A1 and SLC7A1 in cluster 2 and unassigned PPGLs, respectively. These proteins could potentially serve as protein targets, as both play direct roles in cancer biology.

**ATP8A1** (Phospholipid-transporting ATPase IA), which is markedly upregulated in cluster 2) is a catalytic subunit of the druggable heterodimeric P4-ATPase flippase that mediates the translocation of aminophospholipids across biological membranes. The second component of the flippase-cell cycle control protein 50 A (CDC50A, TMEM 30) was also identified in our proteomic analysis among the proteins upregulated in cluster 2 (Fig. 4B). The flippase complex plays a role in cell migration, and overexpression of TMEM30A induces extensive cell spreading and greatly enhances cell migration in Chinese hamster ovary cells, while its depletion has an inhibitory effect [50]. Increased ATP8A1 expression is associated with invasiveness and metastasis in non-small lung cancer (NSLC), and ATP8A1 knockdown decreased the invasiveness of NSCL cancer cells in vitro [51]. In addition, flippase mediates the uptake of synthetic anti-cancer alkyl-phospholipids, such as perifosine and edelphosine (reviewed in [52]), which may, due to flippase upregulation, be effective in cluster 2 PPGL treatment.

**High-affinity cationic amino acid transporter 1** (SLC7A1) is a cell surface protein with 14 putative transmembrane domains that carry arginine, lysine, and ornithine across the plasma membrane. SLC7A1 overexpression has been reported in several cancers, including colorectal tumors [53] and hepatocellular carcinoma [54]. Modulation of SLC7A1 expression via siRNA-mediated knockdown results in cell growth inhibition in CRC and breast cancer cells [55]. More importantly, a monoclonal anti SLC7A1 antibody inhibited the in vivo growth of human colorectal carcinoma tumors in nude mice [56]. Direct functional evidence suggests that SLC7A1 is a potential drug target for PPGLs, with the highest expression in unassigned PPGLs.

### Non-membrane drug targets

In addition to enabling access to otherwise poorly accessible membrane proteomes, the Pitchfork strategy also provides protein expression data on soluble proteins, as it includes the standard “total lysate” proteomic analysis. Markedly upregulated “soluble” cellular enzymes with direct roles in cancer growth also represent potential targets for cell-penetrating LMW inhibitors. Among these soluble proteins, we identified a trio of druggable enzymes upregulated in cluster 1 compared with NAM, all with a pre-established direct role in cancer proliferation.

Mitochondrial **serine hydroxymethyl transferase 2 (SHMT2)** was strongly upregulated in cluster 1 PPGLs, specifically in high—risk tumors driven by *SDHB* pathogenic variants. The SHMT2 protein converts serine to glycine in the mitochondria while producing one-carbon units for the folate cycle, which is important for nucleotide synthesis and the methionine cycle. Serine is the major source of one-carbon units in cancer cells [57, 58] and serine catabolism by SHMT2 is essential for maintaining mitochondrial respiration [59], redox control [60] and proper mitochondrial translation initiation [61]. SHMT2 is induced by HIF1α and MYC during hypoxia to promote cell survival [60].

SHMT2 upregulation in *SDHB*-mutated PPGL may indicate mitochondrial serine catabolism as compensation for the mitochondrial defects caused by SDHB deficiency. Increased SHMT2 protein expression has been reported in several cancer types, including lymphoma, glioma, cholangiocarcinoma, and breast cancer [62]. Higher SHMT2 expression correlates with a worse prognosis or an aggressive phenotype in oral squamous cell carcinoma [63], bladder cancer [64], gastrointestinal tumors [65] and other cancers. Inhibition of SHMT2 activity leads to the reduced proliferation of squamous cell carcinoma cells [66] and prevents the growth of tumors derived from hepatoma cells in a xenograft model [67]. Low-molecular-weight SHMT2 inhibitors SHIN1 and SHIN2 have been developed and shown to have anti-cancer activities against large diffuse B-cell lymphoma [68] opening the way toward experimental evaluation of SHMT2 as a drug target in PPGL.

Mitochondrial **arginase 2 (ARG2)** was also markedly upregulated in C1 tumors, particularly in *SDHB* pathogenic variant tumors. Arginase metabolizes L-arginine to L-ornithine and urea. Arginase 2 overexpression has been observed in several cancers, and its inhibition using LMW inhibitors has been shown to suppress growth or promote apoptosis in various tumor cell lines and animal models [69]. The exact role of arginase 2 in tumor survival remains unknown. However, arginase 2 increases the activity of complex II (succinate dehydrogenase) and regulates HIF-1α [70]. It is therefore tempting to speculate, that similarly to SHMT2, Arginase 2 compensates for the SDHB defect, enabling the survival of *SDHB*-driven PPGL. Arginase inhibitors (nor-NOHA and NOHA) are commercially available [71] making ARG2 a testable drug target for SDHB-related PPGL.

**Autotaxin** is a secreted lysophospholipase that is upregulated in cluster 1, but also in unassigned PPGLs and to a lesser extent in cluster 2 PPGL. Autotaxin produces lysophosphatidic acid (LPA), which in turn signals via six different G-protein-coupled LPA receptors. The autotaxin-LPA axis plays a key role in promoting tumor migration, metastasis, invasion, and angiogenesis [72]. Increased autotaxin expression has been detected in various types of tumors, including glioblastoma, breast cancer, non-small cell lung cancer, and thyroid cancer [72, 73]. Inhibition of autotaxin enzymatic activity by LMW inhibitors has been shown to reduce tumor growth and metastasis in mouse model of breast [74] thyroid [75] and other cancers [72, 73]. A clinical study evaluating the effect of the LMW autotaxin inhibitor IOA-289 in patients with pancreatic cancer was registered at clinicaltrials.gov.

### Limitations of the study

Focusing on high-risk cluster 1 PPGLs, this study included only a limited number of PPGL belonging to cluster 2 and unassigned PPGLs (tumors with no PPGL-related mutations). Samples of cluster 3 PPGLs were not included in the study because of their extremely low incidence and limited availability. The observations made here are based on the analysis of dissected and frozen tumor samples, which represent a highly heterogeneous tumor microenvironment. As such, the increased abundance of the proteins identified here may not reflect the increased abundance explicitly in cancer cells, but may result from their increased expression in the tumor vasculature, associated fibroblasts, immune cells, or other components of the tumor microenvironment.

## Conclusions

In this study, we identified and verified five IMPs and three non-membrane enzymes molecules that exhibited markedly increased expression in cluster 1 PPGLs than in the normal chromaffin tissues, all with already established roles in cancer growth and progression. For all 10 proteins discussed here, experimental LMW inhibitors or monoclonal antibodies are already available, facilitating the evaluation of their therapeutic potential for (cluster 1) PPGL in cell lines and xenograft models. Some cell surface proteins may also be used as targets for tumor imaging radioligands or as theranostic targets for both tumor imaging and treatment. Examples of analogical use can be seen in the upregulated cell surface proteins, PSMA or SSTR2 (already approved as targets for both tumor imaging and therapy), using the same affinity ligand but with radioisotopes of different activities in advanced prostate cancer and neuroendocrine tumors, respectively.

### Electronic supplementary material

Below is the link to the electronic supplementary material.

Additional File 1. Table of all identified proteins (xls file)

Additional File 2. Volacano plots for the Pitchfork methods

Additional File 3. Topology prediction of selected differentially expressed IMPs with mapped peptides identified by the individual Pitchfork methods

## Data Availability

Mass spectrometry proteomics data are available on the ProteomeXchange Consortium via the PRIDE partner repository with the dataset identifier PXD039788. All other data are available from the corresponding authors upon request.

## List of Abbreviations

ACE: Angiotensin-converting enzyme
ANO1: Anoctamin1
ARG2: Arginase 2
ATP8A1: ATPase phospholipid-transporting 8A1
ENTPD1 (CD39): Ectonucleoside triphosphate diphosphohydrolase 1
FDR: False discovery rate
hpTC: High pH-trypsin-cyanogen bromide
IMP: Integral membrane protein
L1CAM (CD171): L1 cell adhesion molecule
LFQ: Label-free quantification, LMW, low molecular weight
LPA: Lysophosphatidic acid
MCAM (CD146): Melanoma cell adhesion molecule
NAM: Normal adrenal medulla
N-glyco-FASP: N-glycopeptide enrichment with filter-aided sample preparation
PGL: Paraganglioma
PHEO: Pheochromocytoma
PPGL: Pheochromocytoma and paraganglioma
PSMA: Prostate-specific membrane antigen
SDHB: Succinate dehydrogenase iron-sulfur subunit, mitochondrial, SHMT2, serine hydroxymethyl transferase 2
SLC7A11: Solute carrier family 7 member 11
SPEG: Solid phase enrichment of N-linked glycopeptides
TCA: Tricarbolic acid
TFA: Trifluoroacetic acid
