# Supplementary material for: Identification of potential molecular targets for the treatment of cluster 1 human pheochromocytoma and paraganglioma via comprehensive proteomic characterization
Source: Clin Proteomics. 2023 Sep 25;20:39. doi: 10.1186/s12014-023-09428-7 (PMC10518975; doi:10.1186/s12014-023-09428-7)

***Additional File 2.*** *Proteomic analysis using Pitchfork strategy. Volcano plots (protein expression fold-change plotted against the statistical significance) separately for each PPGL group (cluster 1, cluster 2, and unassigned PPGLs). Upregulated proteins identified by* ***A****) glycocapture methods (combined N-glyco-FASP and SPEG analyses).* ***B****) hpTC method*

*Colored spots indicate the proteins upregulated (threshold 4-fold, p-val 0.05) in each tumor group than in the control tissues (NAM). Gene names for the corresponding proteins are shown for clarity, and only for the proteins mentioned in the text. The volcano plots include both, IMPs and non-membrane proteins, however, upregulated proteins detected in tumors but absent in NAM could not be plotted (due to the missing fold-change and significance values). Therefore, the number of visualized spots does not correspond with the text, where we discuss only the numbers of IMPs or cell-surface IMPs.*

*
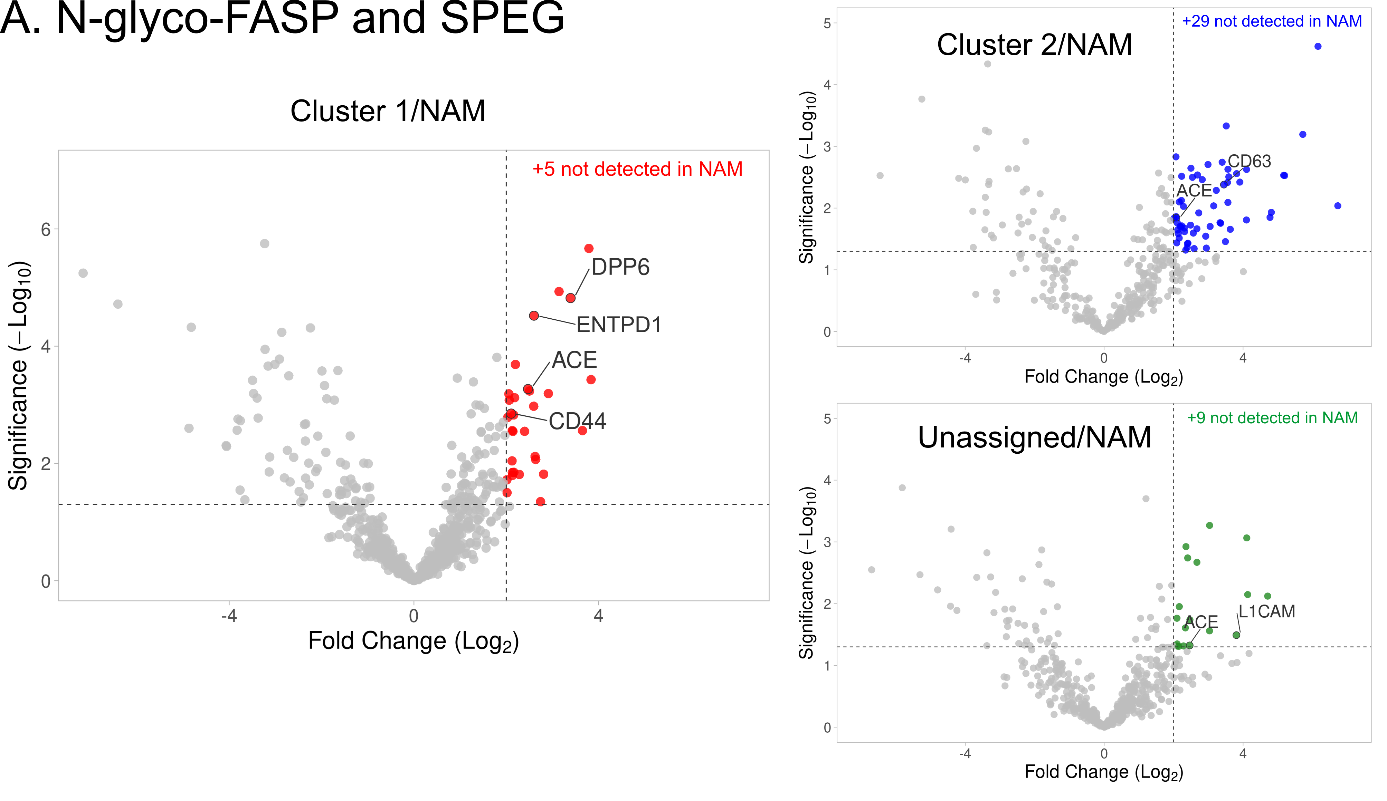
*


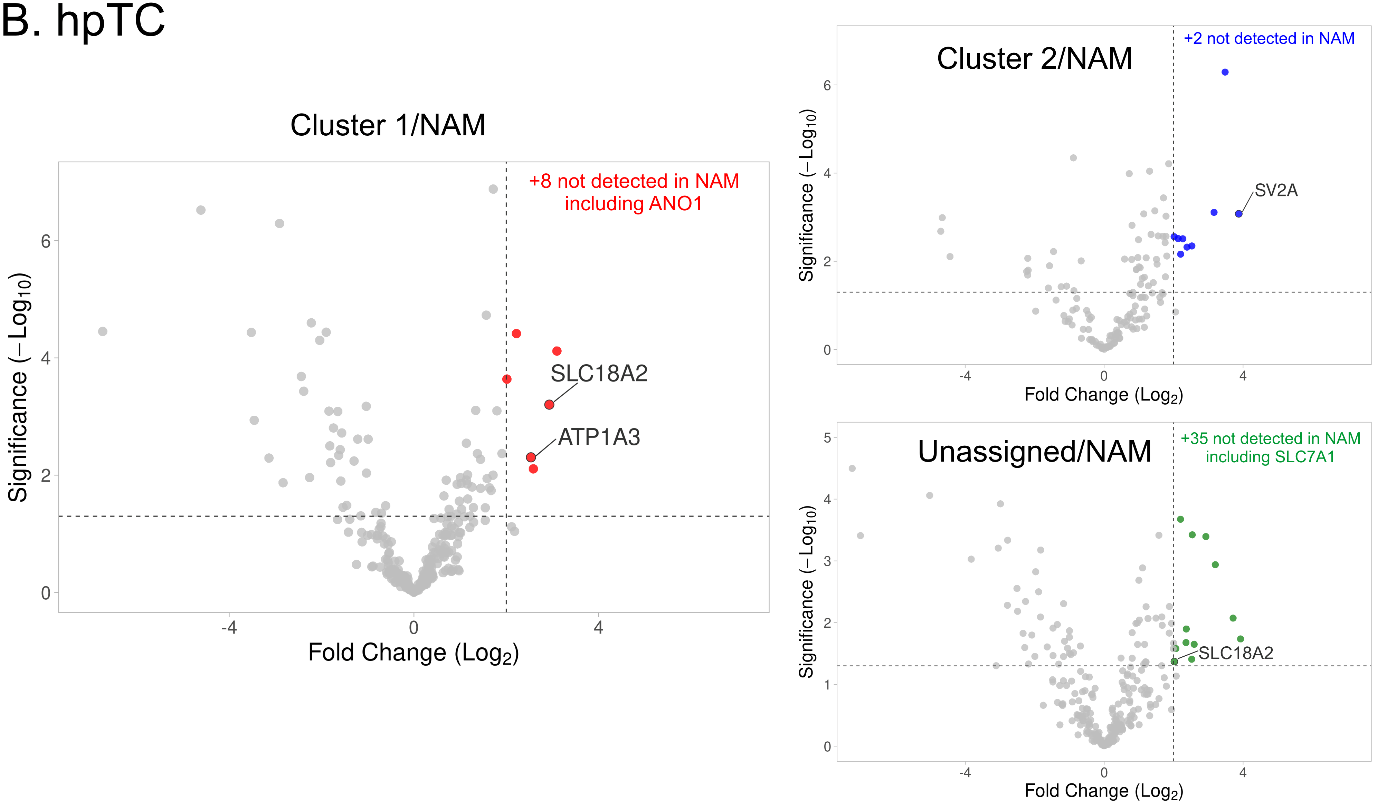

Supplement: Supplementary file 2 — Additional File 2. Volacano plots for the Pitchfork methods [file 12014_2023_9428_MOESM2_ESM.docx]
