# Supplementary material for: Identification of potential molecular targets for the treatment of cluster 1 human pheochromocytoma and paraganglioma via comprehensive proteomic characterization
Source: Clin Proteomics. 2023 Sep 25;20:39. doi: 10.1186/s12014-023-09428-7 (PMC10518975; doi:10.1186/s12014-023-09428-7)

***Additional File 3.*** *Topology prediction of three differentially expressed IMPs with mapping of the peptides provided by hpTC, glyco-capture and the standard proteomic analysis. The prediction was generated by Protter (*<http://wlab.ethz.ch/protter/start/>).


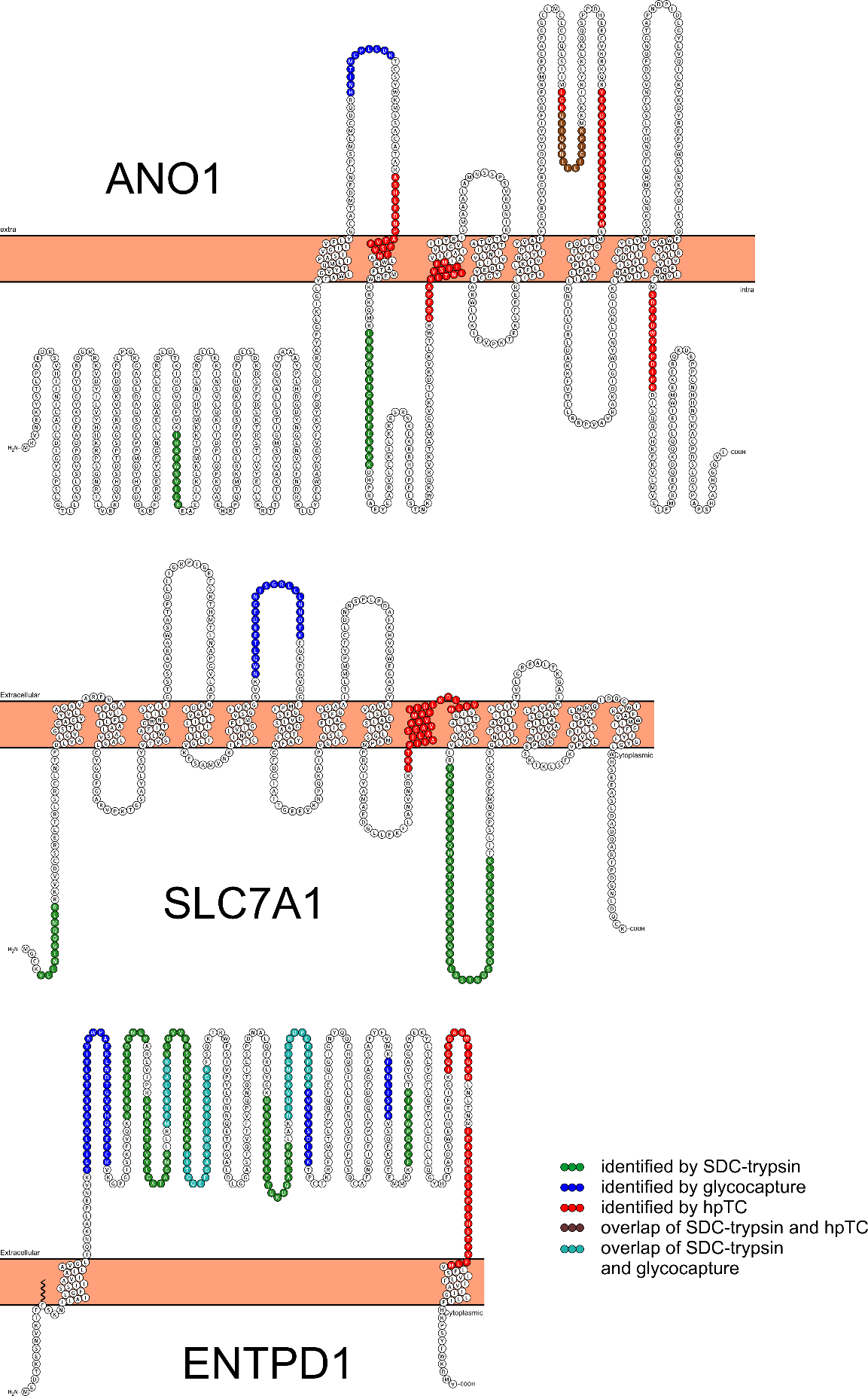

Supplement: Supplementary file 3 — Additional File 3. Topology prediction of selected differentially expressed IMPs with mapped peptides identified by the individual Pitchfork methods [file 12014_2023_9428_MOESM3_ESM.docx]
